# Hot metacognition: poorer metacognitive efficiency following acute but not traumatic stress

**DOI:** 10.1038/s41398-024-02840-z

**Published:** 2024-03-04

**Authors:** Alicia J. Smith, James A. Bisby, Quentin Dercon, Anna Bevan, Stacey L. Kigar, Mary-Ellen Lynall, Tim Dalgleish, Caitlin Hitchcock, Camilla L. Nord

**Affiliations:** 1 MRC Cognition and Brain Sciences Unit, University of Cambridge, Cambridge, UK; 2 Division of Psychiatry, University College London, London, UK; 3 Department of Psychiatry, Herchel Smith Building of Brain & Mind Sciences, Cambridge Biomedical Campus, University of Cambridge, Cambridge, UK; 4 Department of Medicine, Cambridge Biomedical Campus, University of Cambridge, Cambridge, UK; 5 Cambridgeshire & Peterborough NHS Foundation Trust, Cambridge, UK; 6 Molecular Immunity Unit, University of Cambridge Department of Medicine, Cambridge, UK; 7 Melbourne School of Psychological Sciences, University of Melbourne, Melbourne, VIC Australia

**Keywords:** Learning and memory, Human behaviour

## Abstract

Aberrations to metacognition—the ability to reflect on and evaluate self-performance—are a feature of poor mental health. Theoretical models of post-traumatic stress disorder propose that following severe stress or trauma, maladaptive metacognitive evaluations and appraisals of the event drive the development of symptoms. Empirical research is required in order to reveal whether disruptions to metacognition cause or contribute to symptom development in line with theoretical accounts, or are simply a consequence of ongoing psychopathology. In two experiments, using hierarchical Bayesian modelling of metacognition measured in a memory recognition task, we assessed whether distortions to metacognition occur at a state-level after an acute stress induction, and/or at a trait-level in a sample of individuals experiencing intrusive memories following traumatic stress. Results from experiment 1, an in-person laboratory-based experiment, demonstrated that heightened psychological responses to the stress induction were associated with poorer metacognitive efficiency, despite there being no overall change in metacognitive efficiency from pre- to post-stress (*N* = 27). Conversely, in experiment 2, an online experiment using the same metamemory task, we did not find evidence of metacognitive alterations in a transdiagnostic sample of patients with intrusive memory symptomatology following traumatic stress (*N* = 36, compared to 44 matched controls). Our results indicate a relationship between state-level psychological responses to stress and metacognitive alterations. The lack of evidence for pre- to post-stress differences in metamemory illustrates the importance for future studies to reveal the direction of this relationship, and consequently the duration of stress-associated metacognitive impairments and their impact on mental health.

## Introduction

Metacognition refers to our ability to reflect on and evaluate our thinking [1]. The capacity to reflect on past events and evaluate existing knowledge enables us to optimise our predictions about the future, leading to changes in behaviour [2]. In clinical psychology, the metacognitive model of post-traumatic stress disorder (PTSD) [3] highlights the role of maladaptive metacognition in the onset and maintenance of symptoms, as opposed to placing an emphasis on the features of the event memory itself. The model [3] suggests that dysfunctional metacognition about one’s thinking and memory perpetuates cyclical and maladaptive processing of an event, whereby the individual views the self as vulnerable or broken [4], and the world as dangerous and unpredictable [5]. Post-traumatic stress symptoms may arise as a result of an individual believing that their post-event reactions are an indication of mental instability (e.g., “I can’t control my worrying and it is dangerous for me”), and/or can be perpetuated by beliefs that threat monitoring is a necessary coping skill to avoid exposure to another negative event (e.g., “Worrying helps me avoid danger”). Thus, the ability to reflect on whether a particular percept, decision, or memory is accurate or inaccurate—metacognition— is a central component of mental health.

In a laboratory setting, metacognition can be assessed using perceptual, decision-making, and memory tasks that involve making a first-order response (e.g., in a memory context, ‘Have you seen this image before?’), followed by a second-order introspective confidence rating assigned to that response (“How confident are you in your choice?”) [6]. By examining the relationship between task performance and confidence judgements for each decision, we can compute *metacognitive bias*, the tendency for a participant to be under- or over-confident in their decisions, and *metacognitive sensitivity*, the correspondence between subjective confidence and objective task performance. Signal detection curves can then be used to estimate a subject’s *metacognitive efficiency*, controlling for task performance.

There is consistent evidence that distortions in metacognition are linked to diverse problems in mental health, spanning multiple diagnostic categories [7]. Under-confidence in task performance occurs in individuals with a variety of psychiatric disorders, including subclinical obsessive-compulsive disorder (OCD; [8–12]), anxiety [13, 14] and depression [15, 16], while studies of clinical schizophrenia [17–23] and substance use disorder [24, 25] have reported increased confidence in error trials, suggestive of a lack of insight in these populations. The homogeneity of symptoms across different disorder categories could explain some of the overlap in findings whereby metacognitive changes do not fall along disorder lines; instead, they might be better explained by transdiagnostic clusters of symptoms. Using a dimensional approach, Rouault and colleagues [26] found a bi-directional association between metacognition and a transdiagnostic group of symptoms emerging from a factor analysis, labelled ‘compulsive behaviour and intrusive thought’. This symptom factor was linked to overconfidence and poorer metacognition; in contrast, an ‘anxious-depression’ factor was associated with lower confidence and heightened metacognition. Several studies have additionally demonstrated that alterations to confidence are present at the subclinical level [9–11,27, 28] and that confidence abnormalities can normalise after clinical recovery for patients with depression and substance use disorder [15, 29]. While these results highlight an interplay between metacognitive disruptions and mental health symptoms, the mechanisms leading to metacognitive shifts across psychopathology are yet to be established.

The high levels of emotional arousal elicited by negative or stressful events that often precede mental health problems may be one pathway through which metacognition is altered. High arousal is elicited both by trauma (classed as Criterion A in the fifth edition of the Diagnostic and Statistical Manual (DSM-5; [30])) and subclinical stressful events. Specifically, there is evidence that emotional arousal alters the ability to reflect on and monitor cognitive processes, including one’s thoughts, perceptual experiences, decisions and memory [31, 32]. This is mediated by physiological responses to stress: exposure to a highly arousing, negative event activates stress responses governed by the hypothalamic-pituitary-adrenal (HPA) axis [33, 34]. Downstream, the release of cortisol and catecholamines are thought to impact higher-order cognitive functions (e.g., decision-making) via the prefrontal cortex, through binding to glucocorticoid receptors and the subsequent changes to gene transcription [35, 36]. In a recent study, pharmacological manipulation of cortisol (via hydrocortisone administration) caused impairments in perceptual metacognition, independent of subjective stress [37], pointing to a role for physiological state in metacognitive capacity, which may precipitate the effects of negative events on mental health.

Emotional and physiological arousal has also been found to bias episodic memory encoding, although the nature of these disruptions remains controversial [38, 39]. Emotional experiences are generally thought to be recalled better than neutral ones; however, studies have found consistent evidence that not all aspects of memory are affected by emotion in the same way [40–42]. A memory encoding task involving the presentation of image pairs followed by a surprise recognition memory test demonstrated that negative emotion was associated with *item memory* enhancements, but conversely, disruptions in *associative memory* (i.e., memory of links between different items, or items and their associated spatial context). Accordingly, the dual-representation theory [43] suggests that the experience of heightened stress during an event will negatively impact associative memory, disrupting the formation of coherent representations of that event. It is proposed that without the full pairing of the sensory-perceptual details of an event with associated contextual details such as time and location, vivid sensory images of the event involuntarily enter consciousness and are reexperienced in the present as intrusive memories, accompanied by high levels of distress [5].

The effects of arousal on memory are almost always considered separately from the effects of arousal on metacognition. However, under-confidence in one’s perceived memory performance could become self-fulfilling, resulting in increased memory failure and poor clinical outcomes via vicious cognitive-behavioural cycles [44]. Currently, little has been done to explore whether stress leads to biases in metacognition for memory (from here onwards, metamemory), independent of immediate, objective alterations to memory accuracy itself. We aimed to investigate whether a stress induction can produce the impairments to metacognition observed in clinical populations and whether these effects occur independent of changes to objective memory performance. By measuring confidence in performance on the item and associative memory task previously used by Bisby and colleagues [41] in two studies, we investigated whether stress was associated with alterations in metacognition and/or memory (Experiment 1) and whether these changes are present as stable trait-like characteristics in a sample that have experienced traumatic stress (Experiment 2), differing systematically between individuals with clinical levels of intrusive memory symptomology, and controls. Notably, these two hypotheses are not mutually exclusive, and it is plausible that both or neither forms of stress could be associated with metamemory performance.

## Experiment 1

### Methods and materials

#### Participants

Twenty-seven female volunteers without current or previous physical or mental health problems took part (mean age = 26 ± 4.7). Previous research has demonstrated robust individual differences in the magnitude and pattern of inflammatory responses to stress including sex differences [45–47]. Due to funding restrictions limiting our sample size (i.e., we would not have the statistical power to test or control for sex-specific differences), we restricted recruitment for this experiment to females (specifically, individuals assigned female at birth). Other inclusion criteria were (1) 18–35 years old (to limit age-specific effects), (2) normal or corrected-to-normal vision, (3) fluent or native English speakers, (4) no major medical conditions, (5) no current drug treatments, (6) willing to refrain from exercise for 72 h and fast for 12 h preceding the study (the latter three criteria are due to the effects of many medical conditions, drug treatments, exercise, and food on stress response, again aiming to homogenise our sample as much as possible). All participants provided written informed consent prior to taking part. Demographic characteristics are presented in Table 1.

**Table 1:** Sample characteristics for Experiment 1.

	Experiment 1
	Stress induction (*N* = 27)
Gender, number (%)	
Male	0 (0%)
Female	26 (96%)
Non-binary	1 (4%)
Age (years)	
Mean (SD)	26 (±4.7)
Ethnicity, number (%)	
White	23 (85%)
Asian	2 (7%)
Mixed	2 (7%)
Other	0 (0%)
Subjective socioeconomic status (/9)	
Median (IQR)	6 (5–7)

Both experiments described in this paper were approved by the University of Cambridge Human Biology Research Ethics Committee (HBREC.2020.40).

#### Metamemory task

**Fig. 1:**
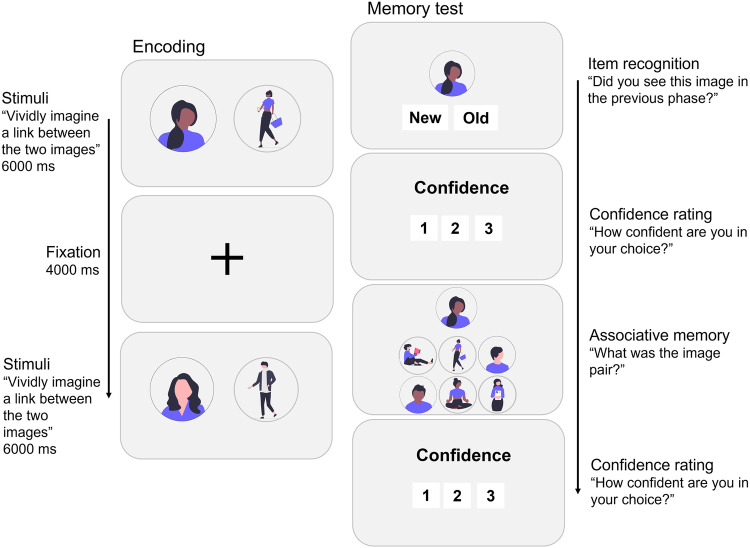
Metamemory task design. In the encoding phase of the task, participants were presented with neutral-neutral, negative-negative, and neutral-negative image pairs and asked whether they could vividly imagine a link between them. A four-second fixation cross was displayed between the presentation of each pair. After a 10-min break, participants returned for a surprise memory test. They were shown one image from each image pair or a new ‘lure’ image and were instructed to judge whether it was new or old. They then rated their confidence from 1 to 3 in their new/old judgement. If the image was selected as old, participants were then asked to select the image pair from a set of 6 images. Again, participants rated their confidence in the selection from 1 to 3. Images used in the task were selected from the International Affective Pictures System (IAPS) and are not presented in Fig. 1.

The original image set used by Bisby et al. [41] was obtained for use in the item and associative memory task. All images had been drawn from the International Affective Pictures System (IAPS; [48]). In the encoding phase of the task (Fig. 1), stimuli were presented in pure neutral (i.e., neutral-neutral images paired together), pure negative (i.e., negative-negative images paired together), and mixed neutral-negative image pairs. Left and right image placement for the neutral-negative trials was counterbalanced. While viewing each image pair, participants were asked whether they perceived each image to be negative or neutral, and whether they could imagine a link between the two images. A four-second fixation cross was displayed between the presentation of each image pair.

After completion of the encoding phase, a 10-minute count-down timer appeared on the screen, ensuring all participants took a short break before the next phase. When the timer reached 10 minutes, participants were able to continue with the task, next taking part in a memory test that included all images from the encoding phase of the experiment (i.e., ‘old’ stimuli), as well as new negative and neutral images added as ‘lure’ stimuli. To assess item memory, one image from each pair or a ‘lure’ image was presented, and participants were instructed to judge whether the picture was old or new. Using a Likert scale, they then rated their confidence from 1 (extremely unsure) to 3 (extremely sure) in their old/new choice. If the item was judged as old, whether correct or incorrect, participants were then instructed to recall the paired associate by selecting the paired image from a choice of three negative and three neutral images displayed below the original image. This part of the task assessed associative memory. Associative memory was tested in one direction with ‘old’ images presented in item memory trials never used at the associative memory level i.e., for a neutral-negative image pair, the neutral image would be presented in the item memory trial, whereas the negative image would be used for the associative memory trial. All images presented in associative memory trials had previously been seen by the participant in the encoding phase of the experiment (were ‘old’ stimuli) to prevent providing any implicit feedback as to whether they were correct or incorrect in their previous item memory judgement i.e., if the participant selected ‘old’ but the image was new, presenting six new images to choose from in the following associative memory assessment may lead to the participant realising that they had made a mistake. After making their selection, they again rated their confidence in their choice from 1–3.

In Experiment 1, after an initial telephone appointment determining eligibility, participants were sent a link to complete the metamemory task from home within one week leading up to their in-person assessment at Cambridge Clinical Research Centre (CCRC) in Addenbrookes Hospital. To ensure engagement with the task, participants were asked to minimise any distractions that may affect their performance. Catch questions were asked at multiple time points to detect inattentive responses. Since participants were required to complete the metamemory task twice in Experiment 1 (pre- and post-stress), all participants were informed by the experimenter, and again in the task instructions, that a memory test would follow the completion of the encoding phase, removing the element of surprise at both time points.

On the day of their in-person assessment at the CCRC, participants undertook a stress procedure (Maastricht Acute Stress Test, MAST; [49]) before completing the metamemory task a second time (employing a different set of images). The MAST and metamemory task were carried out successively as previous studies have shown that peak-stress response occurs after a short delay following a stress induction [46], as well as to avoid the confounding effects of task switching, inattention and/or cognitive load on metamemory performance that may occur if tasks were to be carried out simultaneously [50]. For the encoding phase, a total of 80 trials were presented, consisting of 20 pure neutral, 20 pure negative, and 40 mixed neutral-negative image pair trials. Eighty novel ‘lure’ stimuli were added to the image set for the item memory test.

#### Stress procedure

The MAST is a laboratory stress protocol lasting 12 min that combines intervals of a physical and psychosocial stressor. Participants are required to alternate between immersing their hand in ice water and carrying out mental arithmetic in front of an assessor providing negative feedback (e.g., ‘go faster’). The task has previously been shown to reliably elicit autonomic, glucocorticoid, and subjective psychological reports of stress responses [49].

To confirm whether the procedure reliably induced stress, fasting venous blood samples [51] and scores on the Positive and Negative Affect Schedule (PANAS) scale [52] were recorded prior to (between 08:00 am and 10:00 am) and 45 min after completing the stress induction (representing the time-to-peak stress response [47]). Following the induction, individuals’ subjective stress during the MAST procedure was recorded as a combined score of the perceived stress, pain and unpleasantness experienced during the procedure, each measured on a scale of 0–100.

#### Quantifying item and associative memory performance

For both experiments, our two outcome measures for memory performance were (1) item memory performance, and (2) associative memory performance.

Item memory performance was quantified as *d*’, corresponding to the Z-scored hit rate (“old” responses given for items that are old) minus the Z-scored false alarm rate (“old” responses given to items that are new). In associative memory trials, participants were able to select the correct image, a hit, or the wrong image, a miss, in contrast to item memory trials where false alarms and correct rejections were also possible. Hence, associative memory performance was quantified as the Z-scored hit rate for the recognition of the associated target image.

#### Quantifying metacognitive efficiency

To assess whether metacognitive efficiency was associated with acute stress, we used a generative model of confidence based on signal detection theory (SDT), which estimates the correspondence of confidence ratings to correct or incorrect memory responses and returns a metric of metacognitive sensitivity, meta-*d’* [53]. Using this model, task performance is quantified as *d’ --* memory performance corrected for false alarms. By comparing metacognitive sensitivity (meta-*d’*) to task performance (*d’)*, log(meta-*d’/d’*), a relative measure of metacognitive efficiency is generated. This measure reflects an observer’s tendency for under- or over-confidence in their task behaviour, whilst controlling for fluctuations in performance.

Individual- and group-level posterior distributions for meta-*d’* were estimated in a hierarchical Bayesian manner (HMeta-d [54]) via a freely available toolbox (https://github.com/smfleming/HMM), which uses Markov chain Monte Carlo (MCMC) sampling as implemented in JAGS (http://mcmc-jags.sourceforge.net). This estimation method incorporates subject-level uncertainty in group-level parameter estimates, allowing for direct group comparisons, and performs better than other approaches such as maximum likelihood estimation when there are relatively few trials per subject [54]. Parameter distributions were estimated from 10,000 sampling iterations following 1000 warm-up iterations across each of 3 chains; model convergence was numerically assessed by ensuring that $\hat{R}$ < 1.1 for all parameters.

We additionally used a recent extension of the HMeta-d model, RHMeta-d (fit_meta_d_mcmc_regression.m), which incorporates a simultaneous hierarchical estimation of a regression parameter (beta) that controls variation in logMratio values in relation to a subject-level predictor, in this case, subjective stress (Experiment 1) and score on the intrusions subscale of the IES-R (Experiment 2) [55]. The extended model mitigates issues caused by running post-hoc regressions on hierarchical model parameters such as unwanted shrinkage to the group mean. A sensitivity analysis examining the relationship between subject-level predictors (subjective stress or intrusive memory score) and metacognitive efficiency measured using an alternative subject-level HMeta-d model with post-hoc regression was conducted to assess the robustness of our results and is reported in the Supplementary Materials (Supplementary Fig. S11).

### Results

#### Stress response

Biological measures confirmed the effects of the stress induction on physiological markers of stress (results will be formally reported in a separate manuscript, but our data is openly available and can be accessed here: https://osf.io/jrxf4/). Mean scores of perceived stress, fear and unpleasantness elicited by the MAST induction are presented in Table S3.

#### Item memory performance

**Fig. 2:**
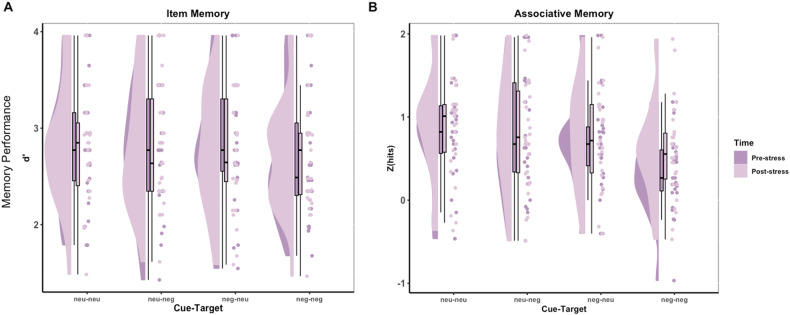
Modified raincloud plots illustrating memory performance in Experiment 1. Memory performance is measured for (**A**) item memory trials, quantified as d-prime (Z(hits) – Z(false alarms)), and (**B**) associative memory trials (Z(hits)), at pre- and post-stress. Individual scatter points show subject-level memory performance estimates for each task, kernel density plots represent the distribution of values and box plots depict medians and interquartile ranges.

Task performance for item memory trials was analysed using a repeated-measures two-way ANOVA with image pair type (neutral-neutral, neutral-negative, negative-neutral, negative-negative) and time point (pre-stress, post-stress) as within-participant factors. We did not find any statistical evidence for a two-way interaction between image pair type and time point (*F*(3,78) = 0.19, *p* = 0.90, η^2^p = 0.01) (Fig. 2A), nor did we find evidence for a statistically significant main effect of either time point (*F*(1,26) = 0.08, *p* = 0.78, η^2^p = 0.003) or image pair type (*F*(3,78) = 0.83, *p* = 0.48, η^2^p = 0.03) on item memory performance (Supplementary Table S1).

#### Associative memory performance

For associative memory performance, we used a repeated-measures two-way ANOVA with image pair type (neutral-neutral, neutral-negative, negative-neutral, negative-negative) and time point (pre-stress, post-stress) as within-participant factors. We did not find any statistical evidence for a two-way interaction between image pair type and time point (*F*(2,57) = 0.87, *p* = 0.43, η^2^p = 0.03) (Fig. 2B), nor a main effect of time point (*F*(1,26) = 1.50, *p* = 0.23, η^2^p = 0.06).

We did however find strong evidence for a main effect of image pair type on associative memory performance (*F*(3,78) = 14.72, *p* < 0.0001, η^2^p = 0.36). To describe this difference further, pairwise comparisons were run between the different image pair types (See Supplementary Table S2). We found strong evidence that associative memory performance was better for neutral-neutral compared to negative-negative image pairs in both the pre-stress task (Bonferroni-adjusted *p* < 0.0001) and the post-stress task (Bonferroni-adjusted *p* = 0.022). We additionally found strong evidence that associative memory performance was better for neutral-negative compared to negative-negative pairs (Bonferroni-adjusted *p* = 0.0004), and better for negative-neutral compared to negative-negative image pairs (Bonferroni-adjusted *p* = 0.0005) in the pre-stress task but not the post-stress task.

#### Metacognitive measures

##### Model-free analyses

We next investigated within- and between-time point differences in metacognitive bias—a model-free metric quantified as mean confidence across both correct and incorrect trials. This measure represents a subject’s tendency to report high or low confidence. Metacognitive bias was calculated for both item and associative memory trials.

We did not find any statistical evidence for differences in metacognitive bias from pre- to post-stress in item memory trials (pre-stress: 2.63 ± 0.30; post-stress: 2.64 ± 0.28; *t*(26) = 0.71, *p* = 0.49) or in associative memory trials (pre-stress: 2.18 ± 0.30; post-stress: 2.20 ± 0.27; *t*(26) = 0.41, *p* = 68). An ordinal logistic regression model relating accuracy to confidence ratings (1, 2 or 3) demonstrated that a 10% increase in accuracy was associated with 36% higher confidence ratings in item memory trials (odds ratio = 3.60, 95% CI = (1.15, 1.41) *p* < 0.0001) and was associated with 60% higher confidence ratings in associative memory trials (odds ratio = 5.95, 95% CI = (1.48, 2.10) *p* < 0.0001), showing that subjects gave higher confidence ratings after correct choices than after incorrect choices (Supplementary Figs. S1 and S2).

Pearson correlations were carried out to examine the relationship between metacognitive bias and item and associative memory performance. We did not find evidence for significant correlations between metacognitive bias and item memory performance (*R* = 0.33, *p* = 0.10; Supplementary Fig. S5A) at pre-stress, or between metacognitive bias and item memory (*R* = 0.31, *p* = 0.11; Supplementary Fig. S5B) or associative memory performance (*R* = 0.33, *p* = 0.10; Supplementary Fig. S5D) at post-stress. We found weak evidence for a positive correlation between metacognitive bias and associative memory performance at pre-stress (*R* = 0.34, *p* = 0.04; Supplementary Fig. S5C).

##### Model-based analyses

Of primary interest was the effect of the stress manipulation on metacognitive efficiency. Estimates were derived from a Bayesian HMeta-d model that required false alarm rate and correct rejection metrics that could not be obtained from associative memory trials. Hence, model-based analyses were conducted on data from item memory trials only.

**Fig. 3:**
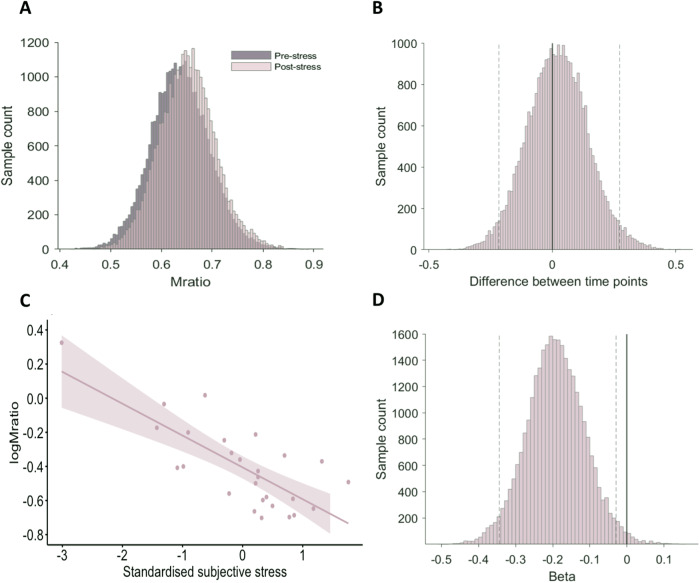
Metacognitive performance estimated by a group-level Bayesian hierarchical model (Fleming^54^). **A** Metacognitive efficiency (µ meta-*d’/d’*) did not differ between pre- and post-stress, as demonstrated by the posteriors of the group-level estimates. **B** The difference in group posteriors of metacognitive efficiency between pre- and post-stress encompasses zero (fixed line) consistent with no evidence of a relationship. Dashed lines represent the 95% HDI (95% HDI = (−0.2156, 0.2729)). **C** Using the RHMeta-d model, higher standardised subjective stress scores predicted poorer logMratio at post-stress. Dashed lines represent the 95% confidence intervals. **D** The distributions of samples over the regression beta parameter, with dashed lines representing the 95% HDI which does not encompass zero (fixed line), is consistent with relatively strong evidence for a relationship between subjective stress and metacognitive efficiency (95% HDI = (−0.3438, −0.0289)).

A generative model assessed the degree to which confidence scores distinguish between correct and incorrect memory judgements (See Methods). We did not find statistical evidence that metacognitive efficiency (the Mratio parameter from the HMeta-d model output) differed between time points (pre- to post-stress) (Fig. 3A, B), with the 95% highest posterior density interval (HDI) wide and including zero (95% HDI = (−0.2156, 0.2729)). Additionally, we did not find evidence for differences in memory performance (*d’*) between pre- and post-stress (Supplementary Fig. S3), nor for correlations between item memory performance and metacognitive efficiency at pre- (*R* = 0.12, *p* = 0.56; Supplementary Fig. S7A) or post-stress (*R* = 0.05, *p* = 0.81; Supplementary Fig. S7B).

There are well-known individual differences in sensitivity to the stress induction [56]. To assess the relationship between the degree of stress subjectively experienced and metacognitive efficiency, we ran a hierarchical regression model (RHMeta-d) on post-stress metacognitive efficiency with standardised post-stress subjective stress responses, as well as age and sex included as covariates. The HDI of each regression beta for sex and age covariates spanned zero and therefore did not provide evidence for significant relationships with metacognitive efficiency (Supplementary Table S6). We found strong evidence for an association between the combined subjective stress measure and metacognitive efficiency (Fig. 3C, D), with the 95% HDI for the regression beta omitting zero (95% HDI = (−0.3438, −0.0289)), suggesting that individuals with heightened psychological responses to stress showed worse metacognitive efficiency.

To explore this relationship further, we re-ran this analysis with the unpleasantness, pain and stress subscales individually. A weak negative relationship was found between metacognitive efficiency and the subjective measure of unpleasantness (Unpleasantness: 95% HDI = (−0.3391, −0.0567) (Supplementary Fig. S8)), however, we did not find evidence for a relationship between metacognitive efficiency and the stress and pain scales, with the 95% beta distributions overlapping zero (Stress: 95% HDI = (−0.2994, 0.0481); Pain: 95% HDI = (−0.2965, 0.0494)). Additionally, we did not find evidence for a relationship between positive and negative affect and metacognition, with beta distributions for both analyses encompassing zero (Negative affect: 95% HDI = (−0.0315, 0.2889); Positive affect: 95% HDI = (−0.2167, 0.1389) (Supplementary Fig. S9)). Intraclass correlation coefficient (ICC) values indicated that the split-half reliability for all measures of metacognition in Experiment 1 was good-to-excellent according to standard reliability interpretations [57] (ICCs from 0.83 to 0.90; See Supplementary Materials S2).

## Experiment 2

In Experiment 2, we sought to explore whether the metamemory disruptions observed for those experiencing a higher level of perceived stress in Experiment 1 were present at a trait level in individuals experiencing stress-related psychopathology. To do so, we applied a transdiagnostic, symptom-based approach, assessing metacognitive efficiency in people with and without current intrusive memories, a clinical phenomenon that occurs after a stressful or traumatic experience for individuals with affective disorders, eating disorders, and anxiety disorders in particular PTSD (Note that 31% of our clinical sample met criteria for PTSD, among other conditions (Table 2)) [58–60]. Methods and predictions for Experiment 2 were pre-registered at https://osf.io/j8de3.

**Table 2:** Sample characteristics for Experiment 2, by group.

	Experiment 2	
	Controls (*N* = 44)	Intrusive memories (*N* = 36)
Gender, number (%)		
Male	12 (27%)	7 (19%)
Female	32 (73%)	29 (81%)
Non-binary	0 (0%)	0 (0%)
Age (years)		
Mean (SD)	47 ( ± 17)	42 ( ± 15)
Ethnicity, number (%)		
White	40 (91%)	32 (89%)
Asian	2 (5%)	1 (3%)
Mixed	1 (2%)	3 (8%)
Other	1 (2%)	0 (0%)
Subjective socioeconomic status (/9)		
Median (IQR)	6 (6–7)	5 (4-6)
IES-R total score (/88)		
Mean (SD)	2.7 ( ± 4.7)	47 ( ± 13)
Intrusions subscale score (/32)		
Mean (SD)	1.2 ( ± 2.0)	20 ( ± 4.8)
Hyperarousal subscale score (/24)		
Mean (SD)	0.45 ( ± 1.3)	11 ( ± 5.8)
Avoidance subscale score (/32)		
Mean (SD)	1.1 ( ± 2.6)	17 ( ± 6.5)
Current DSM-based disorders, number (%)		
PTSD	0 (0%)	11 (31%)
Panic Disorder	0 (0%)	8 (22%)
MDD	0 (0%)	7 (19%)
GAD	0 (0%)	5 (14%)
OCD	0 (0%)	5 (14%)
Agoraphobia	0 (0%)	5 (14%)
Anorexia Nervosa	0 (0%)	4 (11%)
Social Anxiety	0 (0%)	3 (8%)
Phobia	0 (0%)	2 (5%)
AUD	0 (0%)	1 (3%)

*IES-R* Impact of Event Scale- Revised, *PTSD* Post-Traumatic Stress Disorder, *MDD* Major Depressive Disorder, *GAD* Generalised Anxiety Disorder, *OCD* Obsessive-Compulsive Disorder, *AUD* Alcohol Use Disorder.

### Methods and materials

#### Participants

Two power analyses using G*Power 3.1 [61] determined the sample size. To look at the overall effect of emotion on item and associative memory within participants, based on the large effect size previously reported by Bisby & Burgess [40] of η^2^p = 0.63, we calculated that 6 participants would achieve 90% power (alpha = 0.05, G*Power: ANOVA: within factors). However, there is evidence that early studies, on average, produce inflated estimates of effect [62]. Hence, a second power analysis for Experiment 2 was conducted based on a much smaller effect size for the between-subjects effect of clinical phenomenology on memory performance. With a moderate effect size of *r* = 0.30, we calculated that 75 participants would achieve 90% power (alpha = 0.05, G*Power: Linear multiple regression: random model). All participants provided written informed consent prior to taking part.

An initial telephone appointment determined whether each participant met criteria for clinically-relevant levels of intrusions, defined as a score of 12 or above on the intrusions subscale of the Impact of Event Scale – Revised (IES-R; [63]). To complete the scale, the Experimenter required participants to recall a stressful or traumatic event before answering each item with that event in mind, e.g., “I thought about *it* when I didn’t mean to”. Hence, to score 12 or above on the IES-R and be included in the intrusive memories sample, every participant must have experienced an event prior to taking part in the study that they perceived as stressful or traumatic. The Mini-International Neuropsychiatric Interview (M.I.N.I) [64] was administered by trained research staff, under the supervision of a clinical psychologist, to assess the diagnoses of all participants taking part in the experiment. To be included in the control group, participants had to have no current or past mental health problems, assessed using the M.I.N.I.

Inclusion criteria were (1) normal or corrected-to-normal vision, (2) fluent or native English speakers, (3) no major medical conditions, and (4) no current drug treatments. Participants who took part in Experiment 1 were excluded from participation in Experiment 2. All participants took part in the experiment from home and were paid £6/h for their time. The metamemory task was written in JavaScript using the jsPsych framework (v6.2.0; [65]) and the study was hosted on a department server running Just Another Tool for Online Studies (JATOS) v3.3.1 [66].

Of the 105 participants who took part in the experiment, six were excluded due to technical difficulties, thirteen for failing attention-check questions, and two withdrew due to experiencing distress during the task. Sample characteristics are shown in Table 2.

#### Metamemory task

The task itself replicated Experiment 1, with the exception of 200 images presented during the encoding phase and 100 new ‘lure’ images shown during the test. Participants completed the task at one time point. At encoding, participants viewed a total of 100 image pair trials, which consisted of 25 pure neutral, 25 pure negative, and 50 mixed neutral-negative image pair trials, plus 100 new ‘lure’ stimuli added during the item memory test. Participants completed the item-associative memory task at a single time point only. Following consultation with our clinical lead (AB), all high arousal negative images were removed from the set in Experiment 2 to avoid the possibility of causing our clinical sample distress.

#### Deviations from pre-registration

Factor scores on transdiagnostic dimensions generated from the Gillan [67] battery of questionnaires are omitted from this manuscript, due to question items not indexing intrusions pertaining to a traumatic or stressful experience – our primary interest in Experiment 2. All data from the Gillan [67] battery will be made openly available for future analysis (https://osf.io/j8de3).

### Results

#### Item memory performance

We analysed the data from 84 subjects (*N* = 45 controls) who underwent the item and associative memory task. For item memory performance, we used a two-way mixed ANOVA to evaluate the effects of image pair type and group (controls, intrusive memories). Four subjects were identified as extreme outliers and removed from the data set. In our final sample, thirty-six volunteers with clinically relevant levels of intrusions (mean age = 42 ± 15; 29 females) and forty-four volunteers without current or past mental health problems (mean age = 47 ± 17; 32 females) were included in the analysis.

**Fig. 4:**
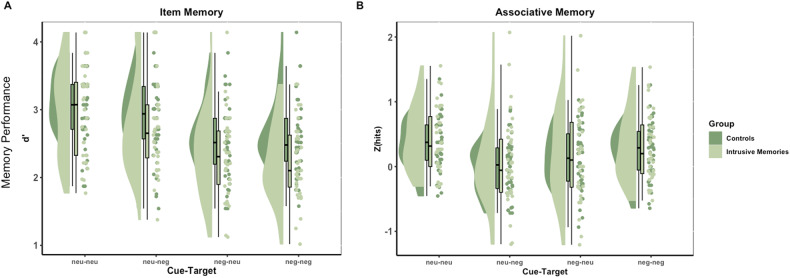
Modified raincloud plots illustrating memory performance in Experiment 2. Memory performance is measured for (**A**) item memory trials, quantified as d-prime (Z(hits) – Z(false alarms)), and (**B**) associative memory trials (Z(hits)), for the control and intrusive memories groups. Individual scatter points show subject-level memory performance estimates for each task, kernel density plots represent the distribution of values and box plots depict medians and interquartile ranges.

Contrary to our pre-registered hypothesis, we did not find evidence for a two-way interaction between image pair type and group (*F*(2,183) = 2.59, *p* = 0.07, η^2^p = 0.03) (Fig. 4A), nor for a simple main effect of group on item memory proportion (*F*(1,78) = 2.57, *p* = 0.11, η^2^p = 0.03). However, there was relatively strong evidence for a simple main effect of image pair type on item memory performance (*F*(2,183) = 46.99, *p* < 0.0001, η^2^p = 0.38).

Due to our pre-registered a priori hypothesis of between-group differences, we also tested the effects of image type separately for the controls and intrusive memories groups.

All simple pairwise comparisons were run between the different image pairs for both the control group and the intrusive memories group (See Supplementary Table S4). For both groups, we found strong evidence that item memory performance (*d’*) was better for neutral-neutral compared with negative-negative image pairs (Bonferroni-adjusted *p* < 0.0001 for both groups), was better for neutral-neutral compared to negative-neutral image pairs (Bonferroni-adjusted *p* < 0.0001 for both groups), was better for neutral-negative compared to negative-neutral (Bonferroni adjusted *p* = 0.002 for the intrusive memories group; Bonferroni adjusted *p* = 0.0007 for the control group), and was better for neutral-negative compared to negative-negative image pairs (Bonferroni-adjusted *p* = 0.0004 for the intrusive memories group; Bonferroni adjusted *p* = 0.013 for the control group). Notably, the simple main effects of image pair type on associative memory performance reported here were contrary to our pre-registered hypotheses. For the intrusive memories group only, we additionally found relatively strong evidence that item memory performance was better for neutral-neutral compared to neutral-negative image pairs (Bonferroni adjusted *p* = 0.01) (note this should not be interpreted as a difference between groups due to the non-significant interaction).

#### Associative memory performance

For associative memory performance, we used a two-way mixed ANOVA to evaluate the effect of image pair type (neutral-neutral, neutral-negative, negative-neutral, negative-negative) and group (controls, intrusive memories). There was no evidence for a two-way interaction between image pair type and group (*F*(3,234) = 0.06, *p* = 0.98, η^2^p = 0.0007) (Fig. 4B), contrary to our pre-registered hypothesis.

We found no main effect of group on associative memory performance (*F*(1,78) = 0.38, *p* = 0.54, η^2^p = 0.01). However, we observed strong evidence for a simple main effect of image pair type on associative memory performance (*F*(3,234) = 25.65, *p* < 0.0001, η^2^p = 0.25) (See Supplementary Table S5), such that associative memory performance was better for neutral-neutral compared to neutral-negative image pairs (Bonferroni-adjusted *p* < 0.0001 for both groups), was better for negative-negative compared to neutral-negative image pairs (control group Bonferroni-adjusted *p* = 0.0001; intrusive memories group Bonferroni-adjusted *p* = 0.014), and was better for neutral-neutral compared to negative-neutral image pairs (control group Bonferroni-adjusted *p* = 0.0008; intrusive memories group Bonferroni-adjusted *p* = 0.009). For the control group, we additionally found relatively strong evidence that associative memory performance was better for negative-neutral compared to neutral-negative image pairs (Bonferroni-adjusted *p* = 0.048). Notably, all simple main effects of image pair type on associative memory performance are contrary to our pre-registered hypotheses.

#### Metacognitive measures

##### Model-free analyses

We next investigated within- and between-group differences in metacognitive bias. Analyses were carried out for both item and associative memory trials for the control and intrusive memories groups.

An ordinal logistic regression model relating accuracy to confidence ratings (1, 2 or 3) suggested that a 10% increase in accuracy was associated with 34% higher confidence ratings in item memory trials (odds ratio = 3.44, 95% CI = (1.15, 1.32), *p* < 0.001) and associated with 39% higher confidence ratings in associative memory trials (odds ratio = 3.91, 95% CI = (1.18,1.55), *p* < 0.001), demonstrating that subjects reported higher confidence ratings after correct choices than after incorrect choices (Supplementary Figs. S1 and S2). We did not find any evidence for between-group differences in metacognitive bias for item memory trials (controls: 2.55 ± 0.34; intrusive memories: 2.54 ± 0.35; *t*(78) = 0.05, *p* = 0.96) nor associative memory trials (controls: 1.75 ± 0.34; intrusive memories: 1.88 ± 0.39; *t*(78) = 1.63, *p* = 0.11).

Pearson correlations were carried out to examine the relationship between metacognitive bias and memory performance. We did not find any statistical evidence for correlations between metacognitive bias and item memory (*R* = 0.18, *p* = 0.28; Supplementary Fig. S6A) or associative memory performance (*R* = 0.11, *p* = 0.49; Supplementary Fig. S6C) for the control group. Conversely, for the intrusive memories group, we found a statistically significant positive correlation between metacognitive bias and both item memory (*R* = 0.49, *p* = 0.002; Supplementary Fig. S6B) and associative memory performance (*R* = 0.39, *p* = 0.02; Supplementary Fig. S6D).

##### Model-based analyses

Using a Bayesian HMeta-d model, we next estimated group-level differences in metacognitive efficiency. As this model requires false alarm and correct rejection metrics that could not be derived from the associative memory trials, model-based analyses were conducted on item memory trials only.

**Fig. 5:**
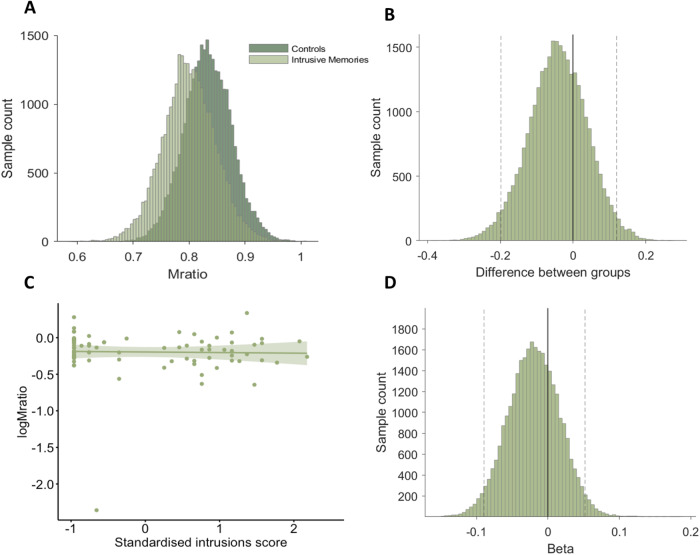
Metacognitive efficiency estimated by a group-level Bayesian hierarchical model (Fleming^54^). **A** Metacognitive efficiency (µ meta-*d’/d’*) was unaffected by clinical intrusive memories, as demonstrated by the posteriors of the group-level estimates for the controls and intrusive memories groups. **B** The difference in group posteriors of metacognitive efficiency between controls and individuals with intrusive memories encompasses zero (fixed line), consistent with no evidence for a relationship between clinical status and metacognitive efficiency (95% HDI = (−0.1988, 0.1192)). Dashed lines represent the two-tailed 95% HDI. **C** Using the RHMeta-d model, no relationship was observed between severity of intrusive memories and logMratio. Dashed lines represent the 95% confidence intervals. **D** The distributions of samples over the regression beta parameter, with dashed lines representing the 95% HDI which encompasses zero (fixed line), demonstrates no relationship between standardised intrusive memory scores and metacognitive efficiency (95% HDI = (−0.0897, 0.0521)), controlling for age and sex.

We first evaluated the correlation coefficient between the intrusive memories and control group within a hierarchical model of meta-*d’*, taking into account uncertainty in subject-level model fits. There was a lack of evidence for any difference in metacognitive efficiency between the control and intrusive memories groups (Fig. 5A, B), with the 95% HDI for the correlation coefficient wide and overlapping with zero (95% HDI = (−0.1988, 0.1192)), contrary to our pre-registered hypothesis. Additionally, we did not find evidence for differences in memory performance (*d’*) between control and intrusive memories groups (Supplementary Fig. S4), nor for correlations between item memory performance and metacognitive efficiency for the control (*R* = −0.28, *p* = 0.06; Supplementary Fig. S7C) or the intrusive memories groups (*R* = −0.05, *p* = 0.77; Supplementary Fig. S7D).

To verify this lack of relationship between metacognitive efficiency and the prevalence of intrusive memory symptoms, we also ran a hierarchical regression model. A regression coefficient, *beta*, was fit within the HMeta-d model to assess the relationship between logMratio and score on the intrusions subscale of the IES-R. The 95% HDI for the regression beta spanned zero suggesting no evidence for a relationship between severity of intrusions and metacognitive efficiency (Fig. 5C, D). We additionally investigated the relationship between metacognitive efficiency and IES-R total scores, as well as the avoidance and hyperarousal subscales using the RHMeta-d model (Supplementary Fig. S10). For all three regressions, the 95% HDI of the beta distribution included zero, again demonstrating a lack of evidence for a relationship between symptoms and metacognitive efficiency (IES-R total 95% HDI (−0.0913, 0.0558); Intrusions 95% HDI (−0.0897, 0.0521); Avoidance 95% HDI (−0.0790, 0.0725); Hyperarousal 95% HDI (−0.1122, 0.0354)). Intraclass correlation coefficient values indicated that the split-half reliability for all measures of metacognition in Experiment 2 was moderate-to-good according to standard reliability interpretations [57] (ICCs from 0.55 to 0.80; Supplementary Materials S2).

Metacognitive analyses using the RHMeta-d model in Experiment 2 are exploratory and go beyond our pre-registration.

## Discussion

Distortions in memory and metacognition occur across psychiatric diagnoses. Stress could contribute to these distortions, reflecting someone’s psychological and physiological sensitivity to pressure from the environment. Indeed, previous studies have reported relationships between stress and metacognition, whereby perceptual metacognition is impaired following hydrocortisone administration [37] and in participants with a greater cortisol response to psychosocial stress [32]. Here, we expand on this in the context of meta-memory, investigating whether stress-associated alterations to metacognition also occur within the memory domain. Using an experimental model of metamemory under stress, we explored whether acute stress was associated with metacognitive alterations in a healthy population. Next, we investigated whether metacognitive alterations were present at a trait-level in a transdiagnostic clinical sample of individuals with intrusive memories. We found mixed evidence for an association between state-level stress and metamemory: at an individual-level, heightened psychological responses to acute stress were associated with compromised metacognitive efficiency, however, no group-level metamemory differences were observed between pre- and post-stress. Contrary to our hypothesis, we found no evidence for metacognitive impairments in our transdiagnostic sample that had experienced traumatic stress, even for those with the highest level of intrusive memories. Additionally, neither the experience of acute nor traumatic stress was associated with differences in item or associative memory.

In Experiment 1, we did not find evidence for pre- to post-stress differences in item or associative memory performance, metacognitive bias or metacognitive efficiency, in contrast to our predictions. Notably, however, participants reporting greater psychological responses to the stress induction (higher reports of ‘unpleasantness’) experienced poorer metamemory in the post-stress memory task. Future studies will be required to establish causation; however, our results may reflect individual differences in reactivity to stress, in line with previous studies [68], highlighting the importance of exploring intra-individual differences that may otherwise be obscured by group-level analyses. Exploratory analyses on the stress sub-scales demonstrated that there was evidence for a relationship between high levels of subjective unpleasantness experienced during the stress procedure and metacognitive efficiency, however, we found no evidence for relationships with the pain and stress subscales, with the 95% HDI of the beta distributions overlapping zero. The heterogeneity of these findings suggests that the link between heightened subjective stress and metacognition may be differentially influenced by sub-components of stress. Future studies will be necessary to confirm the direction of the relationship between stress and metacognitive efficiency. In addition, the stress induction procedure used in this experiment, while well-established in the literature, does not include a “control” condition for the MAST to ensure effects are specific to the stress induction rather than resulting from any task spillover effects; this could be explored in future variations on the stress induction approach. Finally, the lack of significant change in item and associative memory performance between pre- and post-stress in Experiment 1 demonstrates that stress-related impairments to metacognitive efficiency are likely independent of any alterations to memory performance [3].

In Experiment 2, we found no differences in item and associative memory performance, nor in confidence or metacognitive efficiency between individuals with clinical intrusions and those without, in contrast to our hypothesis. Unlike the individual differences captured in Experiment 1, we did not observe any relationship between intrusive memory symptom severity and metamemory. One limitation of this experiment is that chronic stress and/or psychopathology might still affect metamemory at different levels: like most metacognition work in psychiatry, our study related intrusive memory symptoms to *local* metacognition; that is, an individual’s confidence in their memory for the given task. Our results suggest this is unaffected by chronic, intrusive memories. However, recent work by Seow and colleagues [69] emphasised the various hierarchical levels at which metacognition manifests: in contrast to local confidence for single decisions, *global* metacognition reflects broader self-beliefs about one’s abilities and skills and affects evaluations of performance across several decisions and on longer time scales. If global metacognition is more closely related to psychopathology than local metacognition, as this work suggests, future studies should assess this measure in the context of metamemory in people experiencing chronic intrusive memories.

Additional limitations with our experimental setup could be addressed in future studies. The highly arousing negative stimuli used in Experiment 1 were removed from the image set of the metamemory task for Experiment 2 due to the ethical concerns of distressing a clinical sample who were taking part in the study from home due to the COVID-19 pandemic. It remains unclear whether individuals with intrusive memories would have expressed shifts in metamemory in response to the presentation of high-arousal images. Indeed, participants in Experiment 2 performed metacognitively better (control group: Mratio = 0.83; intrusive memories group: Mratio = 0.80) than those in Experiment 1 (Pre-stress: Mratio = 0.63; Post-stress: Mratio = 0.65), which may reflect the procedural differences between the two experiments (use of different stimuli, or from carrying out the task at home).

Other procedural limitations in this study include the limited range (1–3) of the confidence scales used in the metamemory task for both Experiment 1 and Experiment 2. The use of this scale might have restricted the ability to observe smaller deviations in confidence across trials and resulted in the skewed data presented in Fig. S1 (i.e., participants respond correctly and at the highest confidence level for the majority of trials). We highly recommend that future studies use confidence scales with a larger range to ensure that the scale is sensitive enough to measure finer fluctuations in confidence. It should also be acknowledged that in the metamemory task, selecting ‘old’ at the item memory level leads to participants having to complete an additional associative memory judgement. Hence, making an ‘old’ item memory judgement requires more cognitive effort from the participant, which may skew the results towards a higher number of ‘new’ selections. At the associative memory level, no single image choice requires additional cognitive effort, in contrast to item memory responses, making item and associative memory performance less comparable.

Furthermore, in Experiment 2, we aimed to investigate whether individuals experienced trait-level changes to cognition associated with stress following a traumatic event; however, a key limitation to this approach is that we do not have a record of the participants’ physiological response at the time of the event, nor at the time of our testing. Several studies show individual differences in reactivity to stress [68]. Moreover, others show that although subjects may report psychological stress, they do not always present with increased levels of cortisol [70]. Future clinical studies assessing the relationship between stress and cognition should consider taking physiological measurements wherever possible to confirm psychological reports of stress. Finally, while an aim of Experiment 1 was to examine the effect of stress on memory, the stress induction was delivered prior to the encoding phase of the task, limiting our ability to determine whether stress alters memory at the level of encoding or retrieval. Since there was a lack of statistical evidence for any difference in memory performance between the pre- and post-stress task, we suggest that stress did not alter memory at either encoding or retrieval; however, the timing of the stress induction should be considered in future study designs.

A particular strength of our study is the transdiagnostic approach taken. Previous studies have observed distortions to metacognition in the form of under- or over-confidence across psychiatric disorders, demonstrating a lack of clinical specificity; this may reflect a broader issue with the current diagnostic approach [71]. Using the *Diagnostic and Statistical Manual of Mental Disorders* (DSM-5; [30]) criteria, individual psychiatric disorders are heterogeneous and highly correlated with one another. The recent Research Domain Criteria (RDoC) initiative aims to identify transdiagnostic markers of psychopathology that better reflect their psychological traits or underlying cognitive and biological abnormalities [72]. Accordingly, in the current study we used a top-down symptom-based approach to investigate cognitive processes relating to intrusive memories, a transdiagnostic phenomenon that is experienced across affective disorders, eating disorders, and anxiety disorders including PTSD [58–60]. A fundamental challenge with this approach is the ability to identify individuals expressing clinical levels of a single symptom. Currently, this relies on the use of standardised measures that were designed to assess symptoms in accordance with the DSM construct, such as the IES-R, which we used to recruit individuals with intrusive memories in the current study. Hence, the metacognitive processes measured in our sample of individuals in Experiment 2 may be more representative of individuals with PTSD or trauma-related flashbacks than memory intrusions common to other disorders. A crucial aim for future research will be to develop new transdiagnostic measures that can identify clinical symptom clusters that cut across disorders. Alternatively, an intermediate data-driven approach or bottom-up approach working from neurocomputational processes to psychiatric phenomena may be better placed to define more precise phenotypes [73].

In this study, we use “online” measures (assessed using a cognitive task) of metacognitive efficiency and emphasise how the observed findings are in agreement with theoretical accounts (e.g., The Metacognitive Theory of PTSD; [3]) and “offline” self-report measures reported in clinical psychology. Although considerable research has been conducted on metacognition across the various sub-domains of psychology, it remains unclear whether it represents a unitary resource that is applied across different contexts or whether performance is domain-specific [7]. Across fields, most researchers would agree that metacognition allows us to monitor and control our cognitive activity. Despite these conceptual agreements, measures of metacognition differ between the fields of cognitive and clinical psychology, with laboratory-based studies typically using “online” measures that quantify the correspondence between task accuracy and confidence, whereas clinical studies assess “offline” metacognition based on self-report. These self-report measures capture what we ostensibly have explicit access to, requiring retrospection and reflection on self-representations. Recent studies have found that online and offline measures fail to correspond [74], presumably because the nature of “offline” self-report requires an advanced degree of insight and awareness of one’s own metacognitive ability. To gain a better understanding of the correspondence in metacognitive ability across domains and clarify links between metacognition and psychopathology, future research will be required to develop new objective measures that can capture knowledge that isn’t explicitly available to us for clinical research.

To summarise, we demonstrate that greater psychological response to acute stress is associated with poorer metacognitive efficiency, absent of any changes in item or associative memory performance. Understanding the mechanisms that drive distortions in metacognition will support the future development of either cognitive or biological interventions which promote accurate self-evaluation. Recent research has indicated promising avenues for intervention options, such as the use of propranolol to produce noradrenaline blockade and subsequently enhance metacognition (critically, without impacting task performance) [75]. Harnessing an understanding of the mechanisms which underpin the detrimental effects of stress on cognition could therefore enhance the prevention and treatment of psychopathology in the future [70].

### Supplementary information

Supplemental Material

## Data Availability

The data and code used to run the analyses and create the plots in this paper are shared openly on OSF (10.17605/OSF.IO/PGN6U). The pre-registration for Experiment 2 can be found at 10.17605/OSF.IO/J8DE3.
