# Supplementary material for: Hot metacognition: poorer metacognitive efficiency following acute but not traumatic stress
Source: Transl Psychiatry. 2024 Mar 4;14:133. doi: 10.1038/s41398-024-02840-z (PMC10912213; doi:10.1038/s41398-024-02840-z)

**Supplementary Materials**

**Hot metacognition: poorer metacognitive efficiency following acute but not traumatic stress**

Alicia J. Smith, BSc, James A. Bisby, PhD, Quentin Dercon, MSc, Anna Bevan, PhD, Stacey L. Kigar, PhD, Mary-Ellen Lynall, BM BCh PhD, Tim Dalgleish, PhD, Caitlin Hitchcock, PhD^*^, & Camilla L. Nord, PhD^*^

*Joint senior authors.

**S1**: Questionnaires Description

Positive and Negative Affect Schedule – Short Form (PANAS-SF). The PANAS measures positive and negative emotions. The short form comprises of 5 questions on each subscale which are scored on a Likert scale ranging from 0 (very slightly or not at all) to 4 (extremely). Scores on the Positive and Negative Affect subscales range from 0-20, with higher scores representing higher levels of positive or negative affect, respectively.

A visual analogue scale was used to assess subjective stress related to the Maastricht Acute Stress Test (MAST). Participants were asked how stressful, painful and unpleasant they thought the task was on a scale from 0-100 (from ‘not at all’ to ‘very’).

**S2:** Split-half reliability

For Experiment 1, the intraclass correlation coefficient (ICC) value is 0.83 for Mratio (indicating good reliability), with 95% confidence intervals ranging between 0.63-0.92. For meta-d, ICC is 0.87 (indicating good reliability), with 95% confidence intervals ranging between 0.72-0.94. For d’, ICC is 0.90 (indicating excellent reliability), with 95% confidence intervals ranging between 0.79-0.96. Therefore, based on statistical inference, it is appropriate to conclude the level of reliability for results in Experiment 1 to be “good” to “excellent.”

For Experiment 2, the ICC value is 0.55 for Mratio (indicating moderate reliability), with 95% confidence intervals ranging between 0.30-0.71. For meta-d, ICC is 0.77 (indicating good reliability), with 95% confidence intervals ranging between 0.65-0.85. For d’, ICC is 0.80 (indicating good reliability), with 95% confidence intervals ranging between 0.69-0.87. Therefore, it is appropriate to conclude the level of reliability to be “moderate” to “good” for Experiment 2.

**Table S1.** Difference in item memory performance between the image pair types at pre- and post-stress in Experiment 1.

**Table S2.** Difference in associative memory performance between the image pair types at pre- and post-stress in Experiment 1. Results reported in bold represent *p* < 0.05.

|  | negative-negative | negative-neutral | neutral-negative | neutral-neutral |
| --- | --- | --- | --- | --- |
| neutral-neutral | **Pre: *t*(26) = 5.62**  **Post: *t*(26) = 3.18** | Pre: *t*(26) = 1.73  Post: *t*(26) = 1.73 | Pre: *t*(26) = 0.76  Post: *t*(26) = 0.77 |  |
| neutral-negative | **Pre: *t*(26) = 4.72**  Post: *t*(26) = 1.91 | Pre: *t*(26) = 0.48  Post: *t*(26) = 0.38 |  |  |
| negative-neutral | **Pre: *t*(26) = 4.63**  Post: *t*(26) = 1.91 |  |  |  |
| negative-negative |  |  |  |  |

|  | negative-negative | negative-neutral | neutral-negative | neutral-neutral |
| --- | --- | --- | --- | --- |
| neutral-neutral | Pre: *t*(26) = 1.17  Post: *t*(26) = 1.13 | Pre: *t*(26) = 0.07  Post: *t*(26) = 0.58 | Pre: *t*(26) = 1.17  Post: *t*(26) = 1.13 |  |
| neutral-negative | Pre: *t*(26) = 0.63  Post: *t*(26) = 0.81 | Pre: *t*(26) = 0.35  Post: *t*(26) = 0.48 |  |  |
| negative-neutral | Pre: *t*(26) = 1.38  Post: *t*(26) = 0.36 |  |  |  |
| negative-negative |  |  |  |  |

**Table S3.** Descriptive results (mean and standard deviation) of the subjective affect and stress measures recorded at pre- and post-stress for Experiment 1. The final column presents the results of paired sample t-tests applied to evaluate the differences between time points for the PANAS-SF subscales. Significantly lower subjective ratings of negative affect were recorded at post-stress relative to pre-stress. Results reported in bold represent *p* < 0.05.

|  | Pre-stress | Post-stress | Pre- vs. post-stress | |
| --- | --- | --- | --- | --- |
|  | Mean (SD) | Mean (SD) | *t* | *p* |
| *PANAS-SF* |  |  |  |  |
| Positive affect (/20) | 15.11 (4.05) | 13.81 (2.68) | 0.82 | 0.42 |
| Negative affect (/20) | 10.33 (4.10) | 6.52 (1.55) | 4.05 | **0.0002** |
| *Subjective stress* |  |  |  |  |
| Stressful (/100) | NA | 62.67 (20.81) | NA | NA |
| Painful (/100) | NA | 69.30 (17.90) | NA | NA |
| Unpleasant (/100) | NA | 78.26 (19.50) | NA | NA |

**Table S4.** Difference in item memory performance between the image pair types in Experiment 2 for the control and intrusive memories groups. Results reported in bold represent *p* < 0.05.

|  | negative-negative | negative-neutral | neutral-negative | neutral-neutral |
| --- | --- | --- | --- | --- |
| neutral-neutral | **Controls: *t*(43) = 4.97**  **Intrusions:  *t*(35) = 7.67** | **Controls: *t*(43) = 6.35**  **Intrusions:  *t*(35) = 6.49** | Controls: *t*(43) = 1.55  **Intrusions:**  ***t*(35) = 3.41** |  |
| neutral-negative | **Controls: *t*(43) = 3.27**  **Intrusions**:  ***t*(35) = 5.67** | **Controls: *t*(43) = 4.23**  **Intrusions: *t*(35) = 3.96** |  |  |
| negative-neutral | Controls: *t*(43) = 0.82  Intrusions:  *t*(35) = 1.38 |  |  |  |
| negative-negative |  |  |  |  |

**Table S5.** Difference in associative memory performance between the image pair types in Experiment 2 for the control and intrusive memories groups. Results reported in bold represent *p* < 0.05.

|  | negative-negative | negative-neutral | neutral-negative | neutral-neutral |
| --- | --- | --- | --- | --- |
| neutral-neutral | Controls: *t*(43) = 2.33  Intrusions: *t*(35) = 1.49 | **Controls: *t*(43) = 2.33**  **Intrusions: *t*(35) = 3.44** | **Controls: *t*(43) = 7.34**  **Intrusions: *t*(35) = 4.99** |  |
| neutral-negative | **Controls: *t*(43) = 4.81**  **Intrusions: *t*(35) = 3.28** | **Controls: *t*(43) = 2.79**  Intrusions: *t*(35) = 1.69 |  |  |
| negative-neutral | Controls: *t*(43) = 2.02  Intrusions: *t*(35) = 1.76 |  |  |  |
| negative-negative |  |  |  |  |

**Figure S1**. Overall confidence reports for item memory trials, at (A) pre-stress, and (B) post-stress in Experiment 1, and for both (C) the intrusive memories group, and (D) the control group in Experiment 2. Error bars represent standard deviation. Across trials, confidence reports were reliably related to memory accuracy, with participants reporting higher confidence ratings for correct than incorrect responses.


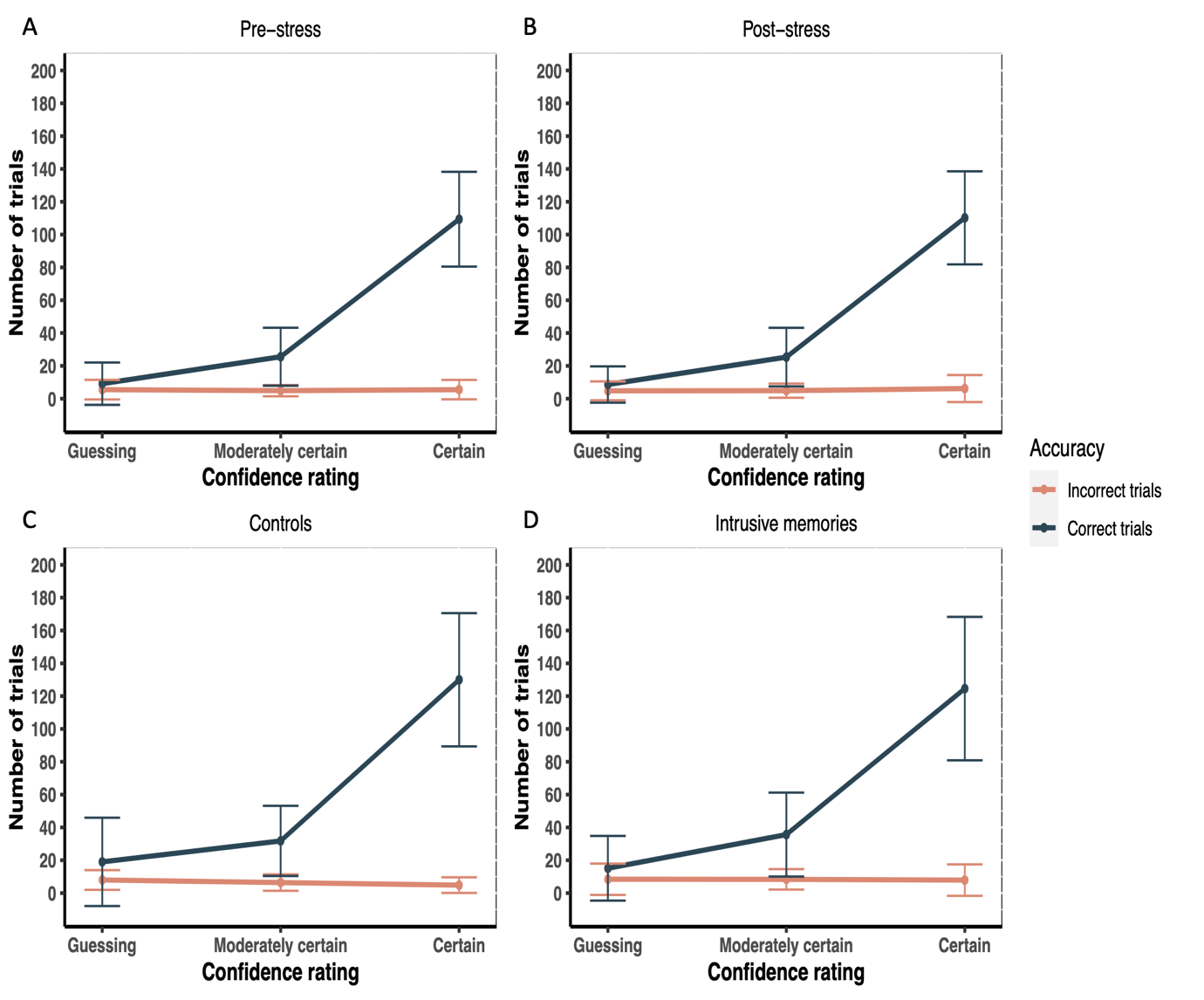


**Figure S2**. Overall confidence reports for associative memory trials at (A) pre-stress, and (B) post-stress in Experiment 1, and for (C) the intrusive memories group, and (D) the control group in Experiment 2. Error bars represent standard deviation. Across trials, confidence reports were reliably related to memory accuracy, with participants reporting higher confidence ratings for correct than incorrect responses.


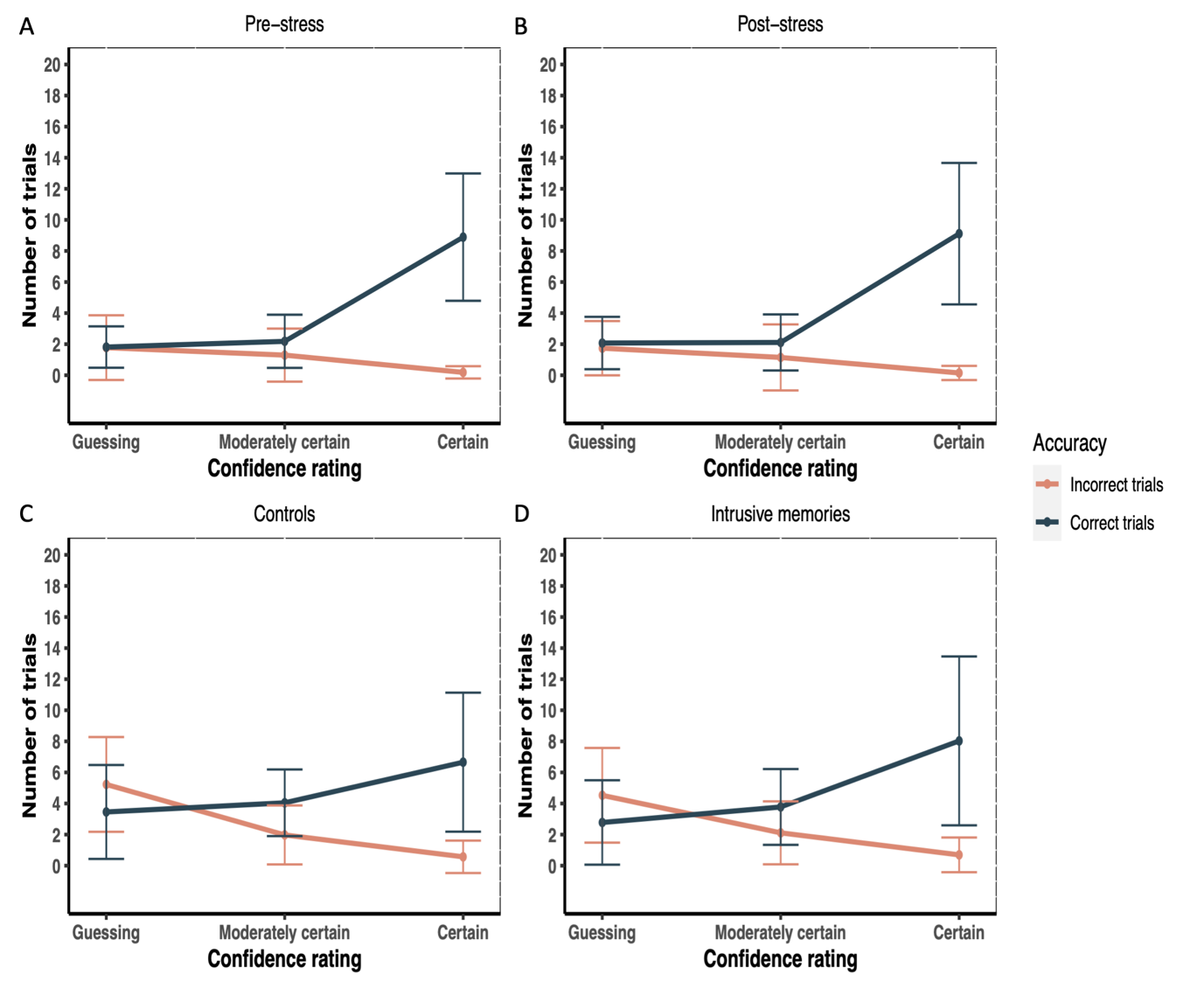

**Figure S3.** Mean memory performance for item memory (d’) and associative memory trials (Z(hits)) for Experiment 1.

**Figure S4.** Mean memory performance for item memory (d’) associative memory trials (Z(hits)) for Experiment 2.

**Figure S5.** Correlation between item memory performance (*d’*) and metacognitive bias at (A) pre-stress, and (B) post-stress, and between associative memory performance (Z(hits)) and metacognitive bias at (C) pre-stress and (D) post-stress, in Experiment 1.


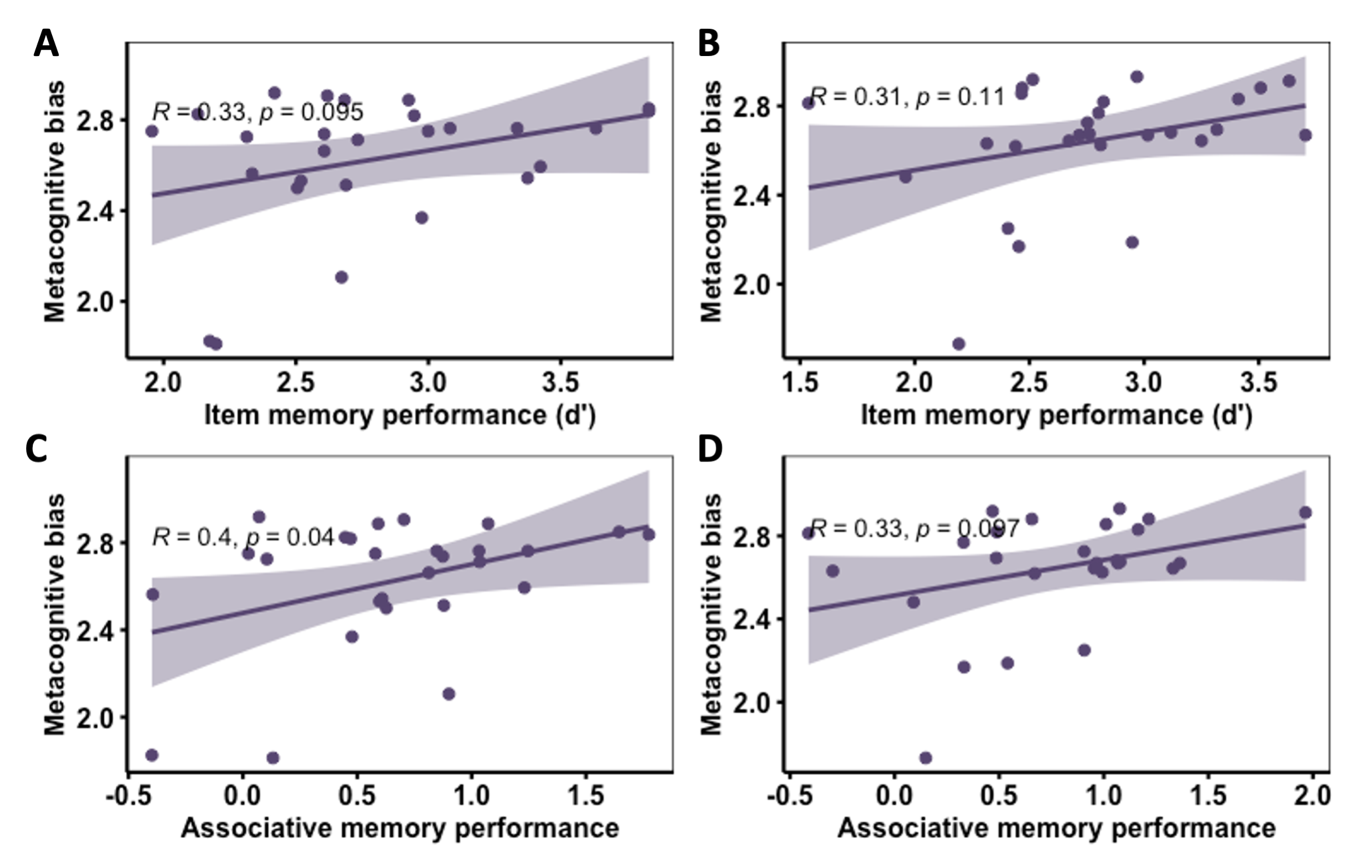


**Figure S6.** Correlation between item memory performance (*d’*) and metacognitive bias for (A) the control group, and (B) the intrusive memories group, and between associative memory performance (Z(hits)) and metacognitive bias for (C) the control group and (D) the intrusive memories group, in Experiment 2.


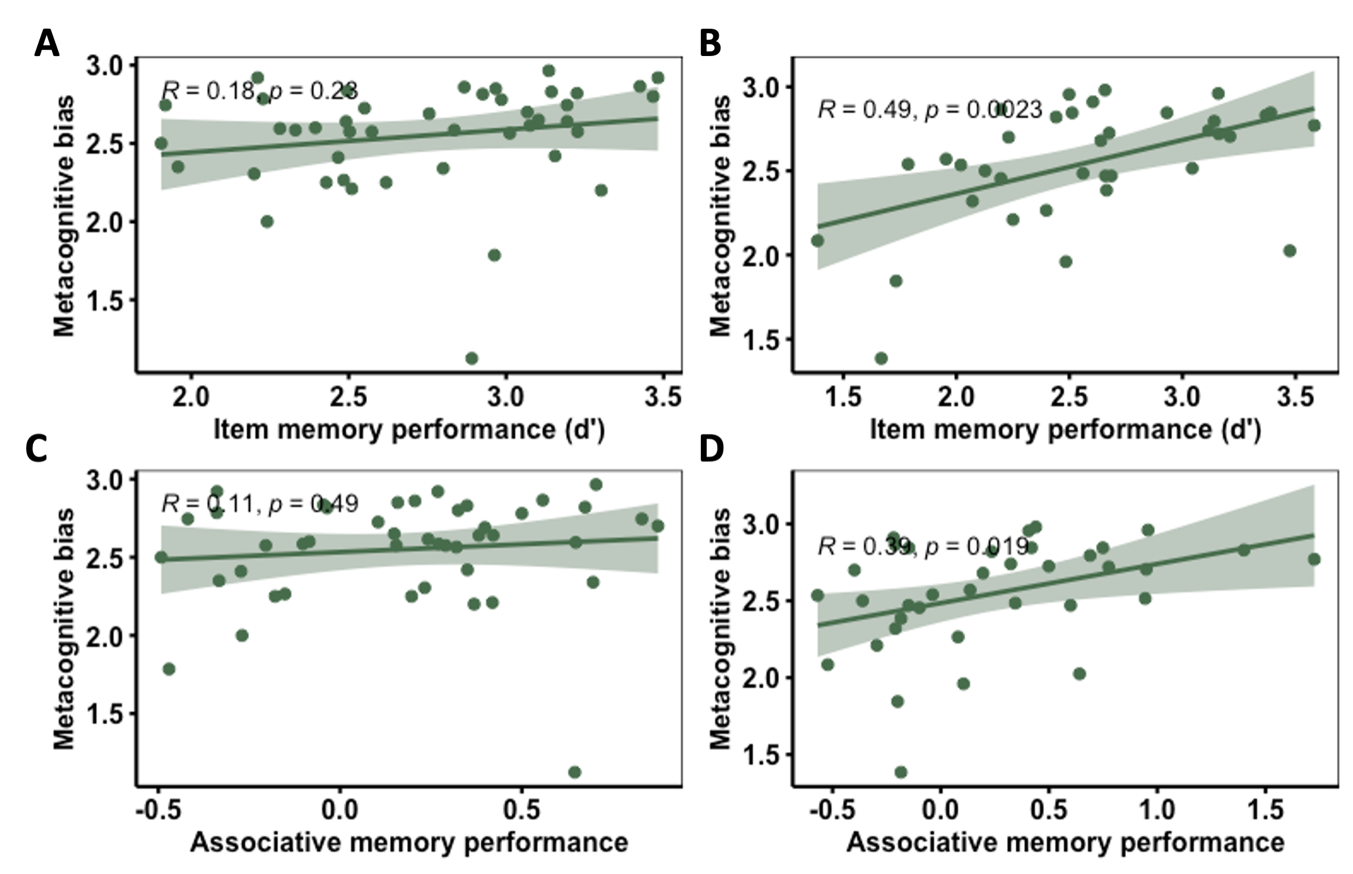


**Figure S7.** Relationship between item memory performance (*d’*) and metacognitive efficiency measured in Experiment 1 at (A) pre-stress, and (B) post-stress, and in Experiment 2 for (C) the controls, and (D) the intrusive memories groups.


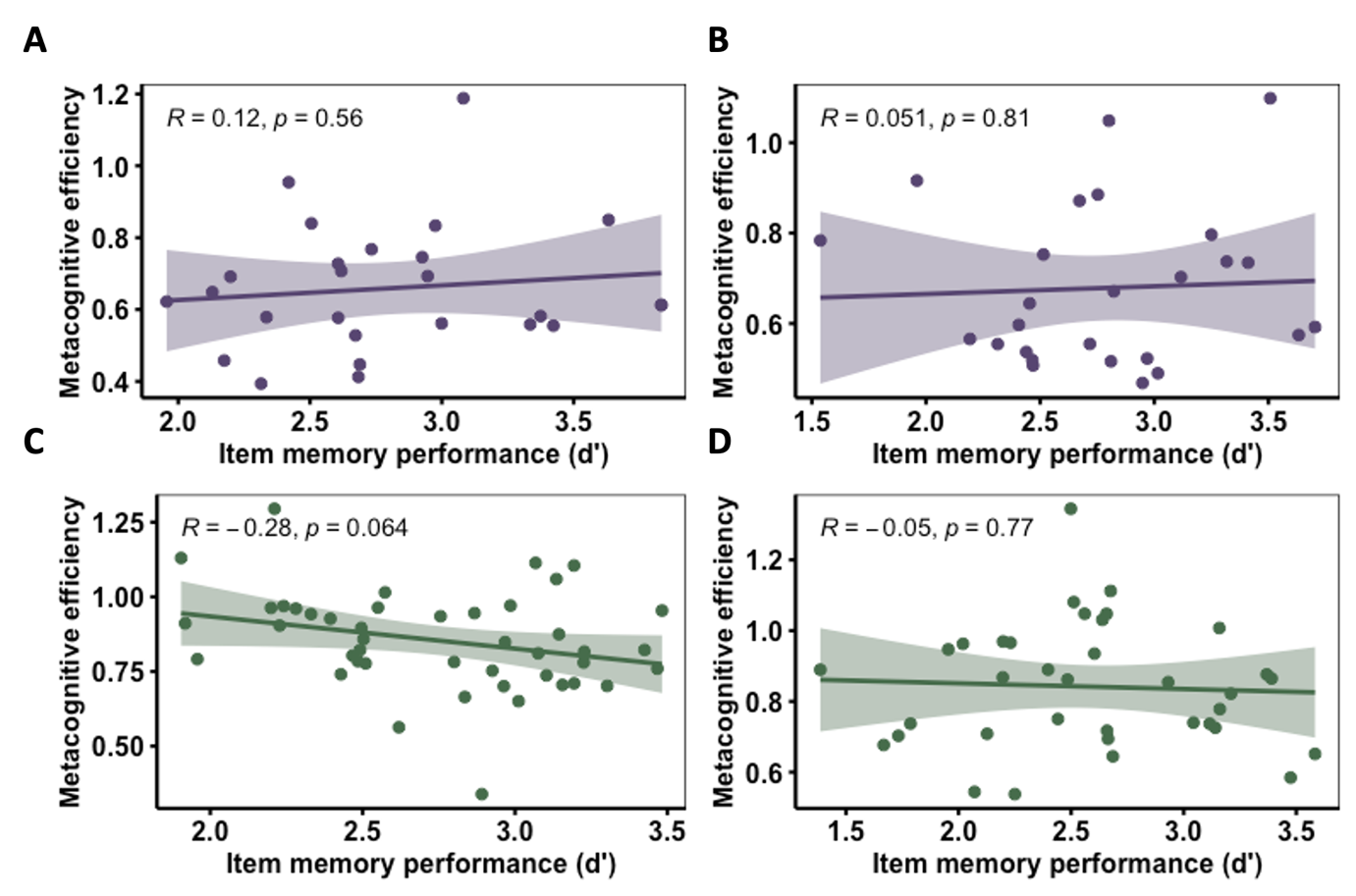


**Figure S8.** Metacognitive efficiency measured using the RHMeta-d model, for relationships between metacognitive efficiency at post-stress and each of the three subjective stress subscales (Unpleasantness (A&B): 95% HDI = (-0.3391, -0.0567); Stress (C&D): 95% HDI = (-0.2994, 0.0481); Pain (E&F): 95% HDI = (-0.2965, 0.0494)).


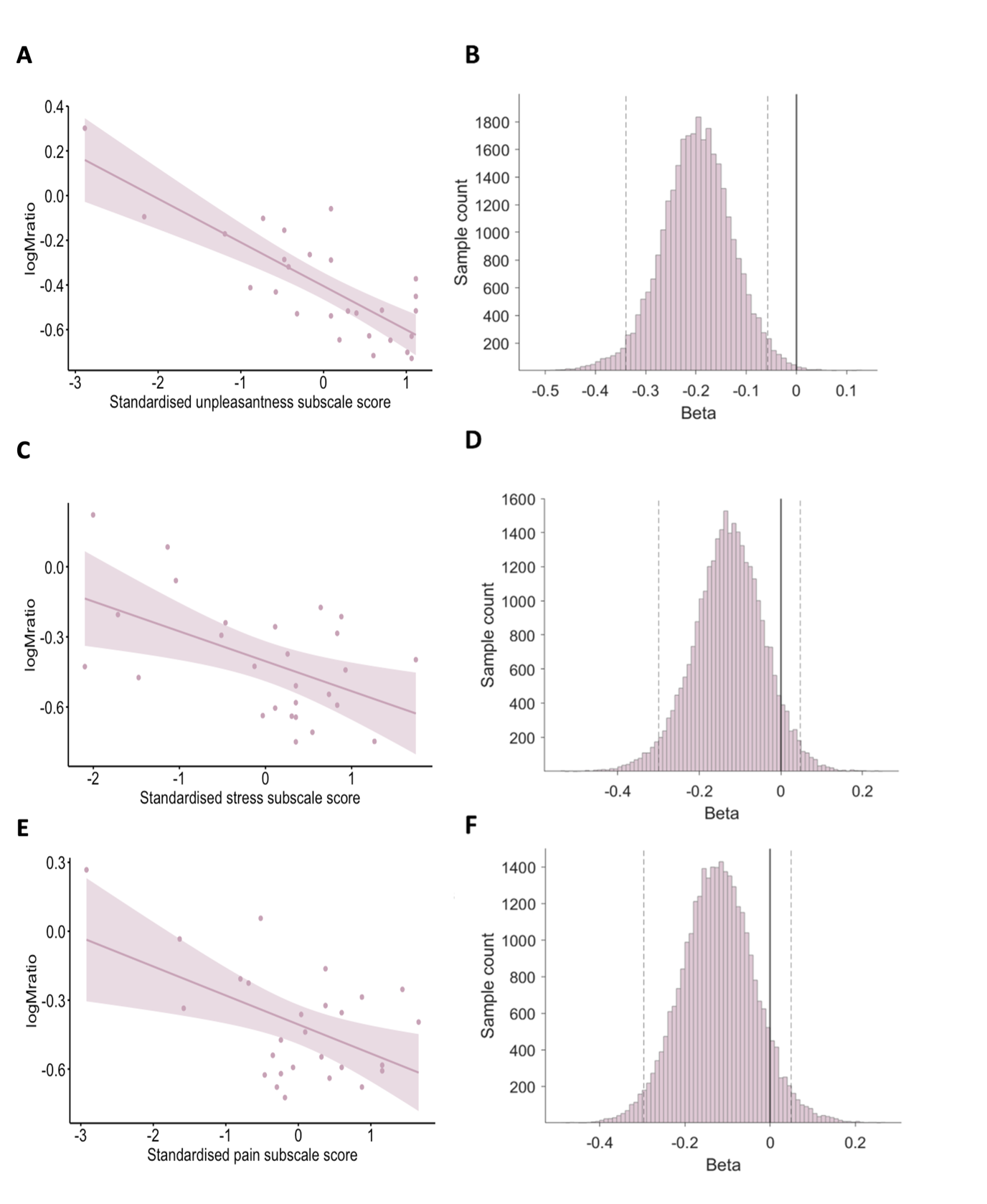


**Figure S9.** Metacognitive efficiency measured using the RHMeta-d model, to investigate relationships with scores on the negative and positive affect subscales of the Positive and Negative Affect Schedule (PANAS) at post-stress (PANAS negative affect (A&B): 95% HDI = (-0.0315, 0.2889); PANAS positive affect (C&D): 95% HDI = (-0.2167, 0.1389))

**
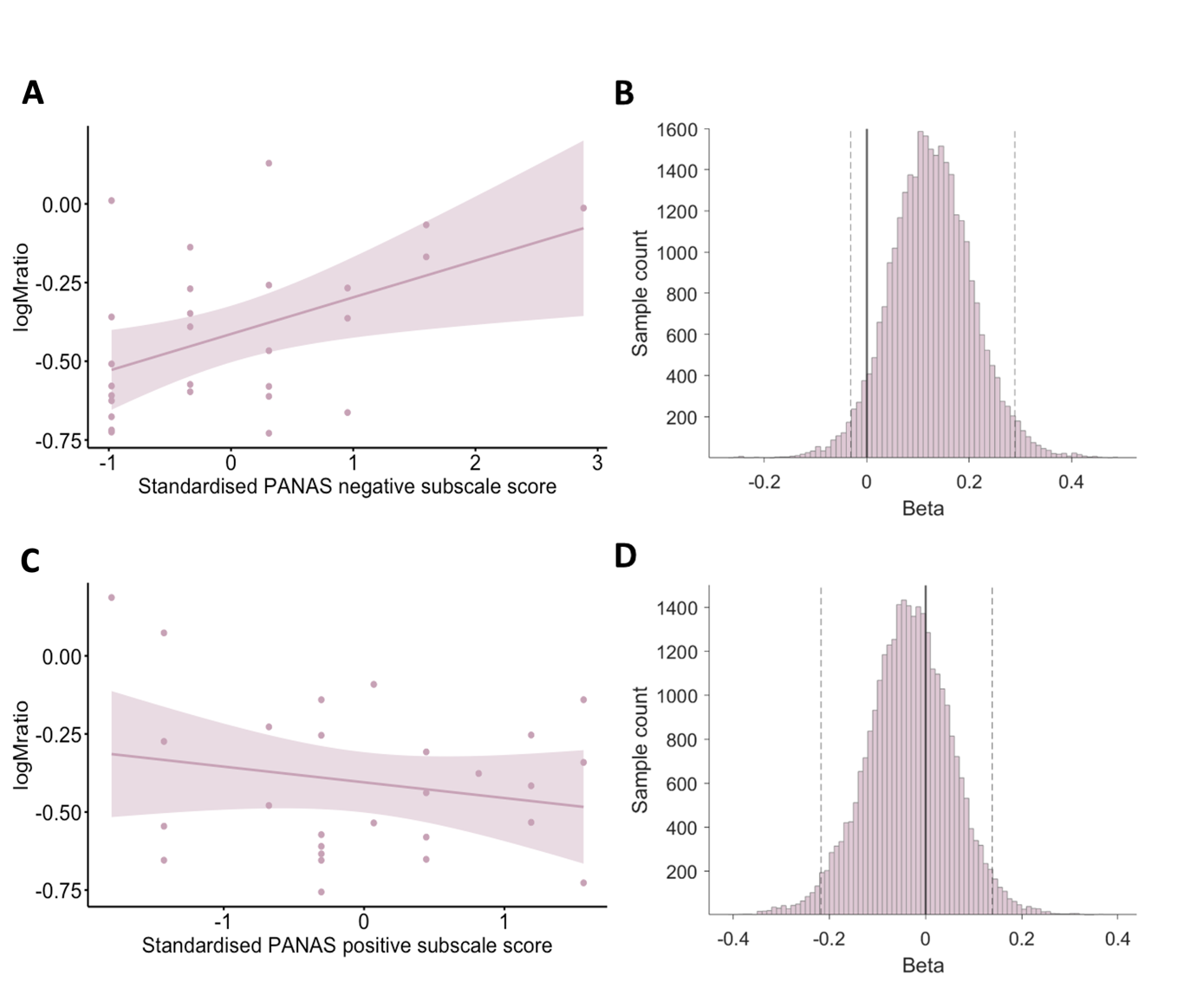
**

**Figure S10.** Metacognitive efficiency measured using the RHMeta-d model, to assess relationships between Mratio and total scores on the Impact of Event Scale – Revised (A&B: IES-R total 95% HDI (-0.0913, 0.0558)), as well as scores on each of the subscales individually (C&D: Intrusions 95% HDI (-0.0897, 0.0521); E&F: Avoidance 95% HDI (-0.0790, 0.0725); G&H: Hyperarousal 95% HDI (-0.1122, 0.0354)), with age and sex added as covariates.


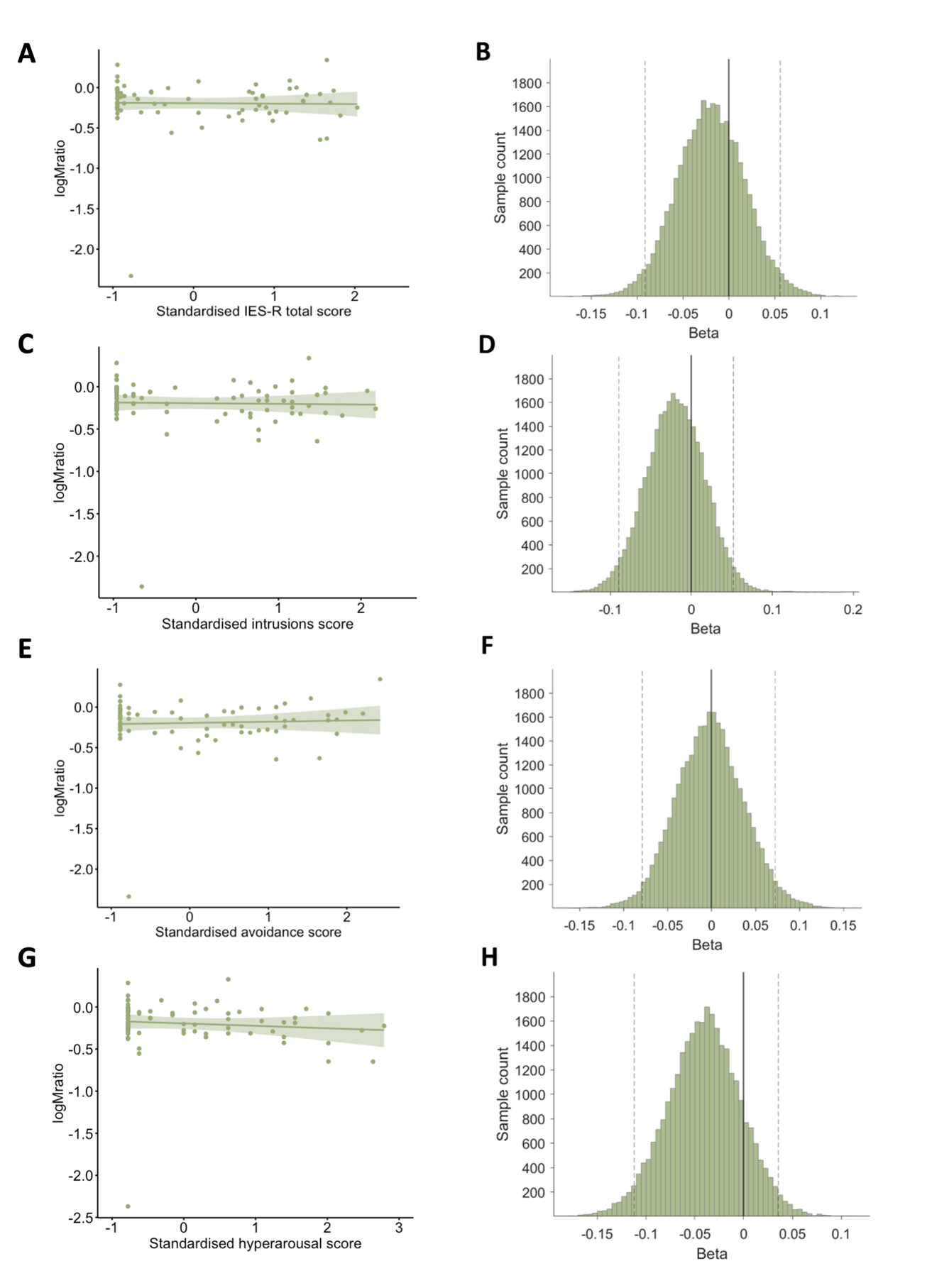


**Table S6.** Mean sampled beta and 95% HDI for covariates included in the RHMeta-d models for Experiment 2.

| Covariate | Mean sampled beta | HDI^a^ |
| --- | --- | --- |
| *IES-R* |  |  |
| Age | 0.0012 | (-0.0029, 0.0050) |
| Sex | -0.1170 | (-0.2793, 0.0455) |
| *Intrusions* |  |  |
| Age | 0.0014 | (-0.0032, 0.0057) |
| Sex | -0.0974 | (-0.2744, 0.0840) |
| *Avoidance* |  |  |
| Age | 0.0014 | (-0.0025, 0.0054) |
| Sex | -0.1175 | (-0.2781, 0.0432) |
| *Hyperarousal* |  |  |
| Age | 0.0009 | (-0.0032, 0.0051) |
| Sex | -0.1053 | (-0.2636, 0.0713) |

^a^ Sampled beta 95% Highest Density Interval

**Figure S11.** Sensitivity analysis. Metacognitive efficiency measured using a single-subject level HMeta-d model with a post-hoc regression to assess relationships between Mratio and (A) subjective stress in Experiment 1 (b = -0.12, t(25) = -2.34, *p* = 0.03), and (B) intrusive memory severity in Experiment 2, with age, sex and their interaction added as covariates (b = -0.25, t(72) = -1.06, *p* = 0.29). Findings replicate those produced using the RHMeta-d model, reported in the main manuscript.


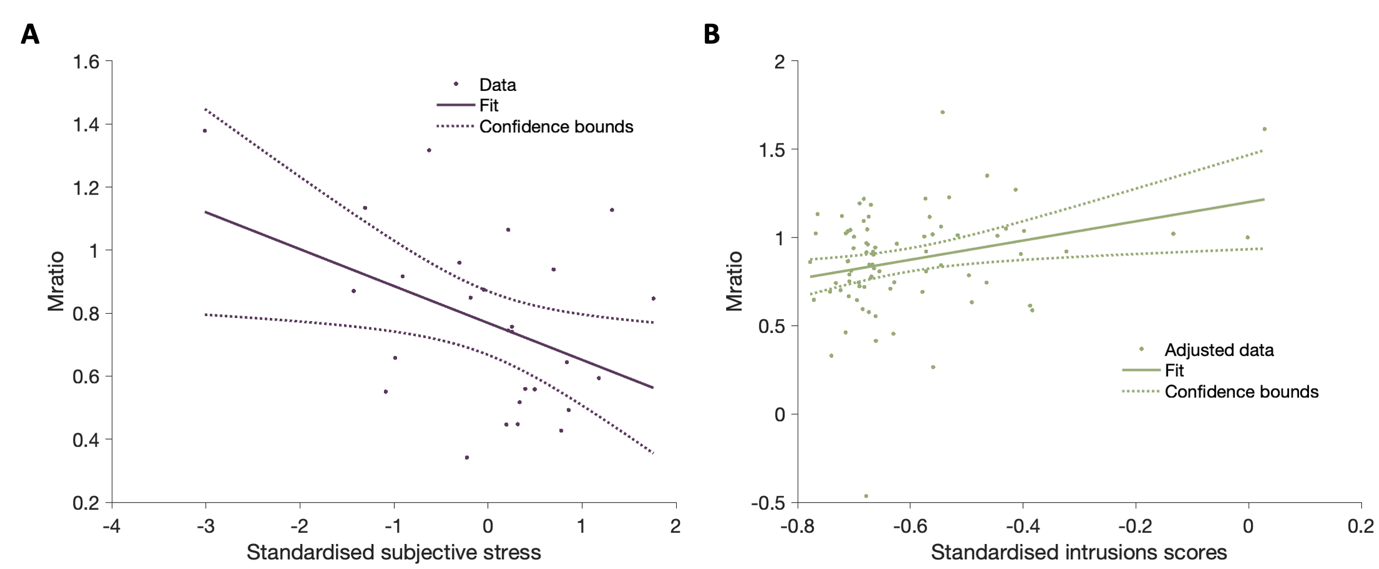

Supplement: Supplementary file 1 — Supplemental Material [file 41398_2024_2840_MOESM1_ESM.docx]
